# Performance evaluation of a novel adjustable lampshade-type reflector (ALR) in indoor farming practice using choy sum (*Brassica rapa* var. *parachinensis*)

**DOI:** 10.3389/fpls.2022.1057553

**Published:** 2023-02-10

**Authors:** Jim Junhui Huang, Zijie Guan, Xiaotang Hong, Weibiao Zhou

**Affiliations:** ^1^ Environmental Research Institute, National University of Singapore, Singapore, Singapore; ^2^ Department of Food Science and Technology, National University of Singapore, Singapore, Singapore

**Keywords:** adjustable lampshade-type reflector, choy sum, biomass, morphology, pigments, antioxidant capacity

## Abstract

The retrieval of lost light energy for promoting vegetable development could be a challenge in indoor farming practice, yet little is attempted so far. In this study, the performance of a novel adjustable lampshade-type reflector (ALR) was investigated to evaluate the feasibility of applying such a device in indoor farm racks (IFR). This application targeted at reflecting stray light back to the IFR for improving the growth and quality of leafy vegetable choy sum (*Brassica rapa* var. *parachinensis*). The optimal configuration of ALR was firstly confirmed *via* simulations using TracePro software. The combination of an included angle at 32° and a reflective board width of 10 cm, under 12 cm of distance between the light sources and the germination tray surface, was revealed to achieve a cost-optimal reflective effect. The simulation-based ALR was subsequently custom-built for actual performance validation. It was shown to effectively produce uniform distributions of temperature, relative humidity, and photosynthetic photon flux density as well as to accumulate more photosynthetic photon energy density along the cultivation shelf. Compared with the control where no ALR was used, the fresh weight and the dry weight of choy sum shoots cultivated using an ALR were increased by up to 14% and 18%, respectively. In addition, their morphological traits were found to be more uniform. Furthermore, their total carotenoid level was enhanced by up to 45%, while the chlorophyll *b* level was markedly decreased. However, no statistically significant difference was found in total phenolic content and antioxidant capacity across the shelf, indicating that the ALR application led to a more uniform antioxidant-related quality of choy sum shoot. ALR application in IFR can thus effectively boost vegetable production and result in quality improvements under an identical amount of electricity consumption in indoor farming compared with ALR-free control.

## Highlights

▪ The optimal combination of ALR included angle and reflective board width was simulated.
▪ ALR significantly increased the fresh weight of choy sum shoot by up to 14%.
▪ ALR markedly enhanced the dry mass of choy sum shoot and total leaf by up to 24%.
▪ ALR remarkably elevated the total carotenoid level in choy sum shoot by up to 45%.

## Introduction

Over decades, the over-development of urbanization in conjunction with a rapid increase in population has resulted in a significant reduction of arable land area per capita (hectares per person) in the world, which called for a bigger demand on common food such as grains and vegetables. This situation urged people to develop efficient methods to produce more food (Satterthwaite et al., 2010; Benke and Tomkins, 2017). Vegetables, especially leafy green vegetables, have been shown to present a variety of bioactive metabolites such as carotenoids, phenolic compounds, vitamins, and glucosinolates that could benefit human health and supplement daily nutritional needs (Li et al., 2018; Liang et al., 2018; Lee et al., 2020; Huang et al., 2021a; Du et al., 2022). Vegetables are traditionally cultivated by using outdoor farming methods such as the conventional flat planting approach. Nevertheless, the disadvantages of those traditional outdoor farming methods, such as unstable solar radiation and weather conditions, low land utilization rate due to flat planting, and pest invasion, make them inefficient (Buttar et al., 2006; Alvino and Barbieri, 2016; Ngosong et al., 2021). In recent years, indoor vertical farming has been gradually developed and become a more attractive approach to grow vegetables indoors by eliminating the above-mentioned limiting factors that hamper agricultural production. Among others, the most well-known lighting source currently applied in indoor farming is light-emitting diode (LED) due to its higher energy conversion efficiency and longer operating life span compared with other lighting sources (Despommier, 2013; Benke and Tomkins, 2017; Miao et al., 2019).

The setting of indoor farm racks (IFR) is among the basic devices for implementing vertical planting. It includes multiple shelves/storeys arranged in a vertical manner and/or equipped with lighting panels/groups as well as irrigation systems. This maximizes space utilization in all three dimensions, thereby dramatically increasing the productivity of an indoor farm (Benke and Tomkins, 2017; Kozai et al., 2019). It is noteworthy that the heterogeneous distribution of light on IFR cultivation shelves is always an ineluctable issue to be addressed (Asiabanpour et al., 2018). However, the common designs of IFR hardly consider the light distribution issue along a cultivation shelf. A significant amount of light ray originated from the lighting sources installed at the margins or even in the middle of a shelf is lost to the environment rather than being directed to the shelf (Kozai, 2019; Kim et al., 2021). In addition, IFR and its shelves also face issues of environmental variability, such as the uneven distribution of temperature and humidity (Akiyama and Kozai, 2016; Tsitsimpelis et al., 2016; Jiang et al., 2018; Sabudin et al., 2022), which may further lead to a jagged morphology of the seedlings grown on them. Thus, an efficient solution could be the application of a reflector or reflective device with a lampshade configuration in IFR.

The feature development of lampshade-type reflectors underwent several key stages such as those with un-adjustable planar reflective board (fixed shapes and angles) (Armstrong, 1978; Michaloski, 1991), those with adjustable planar reflective board but only applicable to a fixed configuration of lighting sources (Dumont, 2013), as well as those with adjustable curved/arched reflective boards (Chelf, 2002; Cronk, 2007; Keen, 2011). To the best of our knowledge, however, so far there has been no reflector that fully considers and combines all key features such as reflective boards that are adjustable in a wide range, structures applicable to multiple and different configurations of lighting sources, as well as maintenance of ventilation.

In this study, a novel adjustable lampshade-type reflector (ALR) was proposed, designed, built, and validated. This invention aims to redirect stray light rays towards desirable locations. The idea is to retrieve originally lost light energy for promoting vegetable development and consequently decrease vegetable production cost. In addition, it also tries to combine all the above-mentioned features of reflectors. To evaluate the efficiency of such a reflector, TracePro software was firstly applied to simulate and design ALR’s optimal configuration to yield the desired performance outcome. The ALR-regulated distribution of environmental factors such as temperature, relative humidity (%RH), photosynthetic photon flux density (PPFD, 400–700 nm), and photosynthetic photon energy density (PPED) across the cultivation shelf was then tested under selected included angle of the reflective board. Choy sum (*Brassica rapa* var. *parachinensis*), a widely cultivated leafy green vegetable in Asia, was finally adopted as the target plant for comparison in terms of its growth and quality with and without ALR application. It was hypothesized that this ALR could efficiently improve the biomass and key quality attributes of choy sum seedlings when appropriately applied.

## Materials and methods

### Adjustable lampshade-type reflector

**Figure 1:**
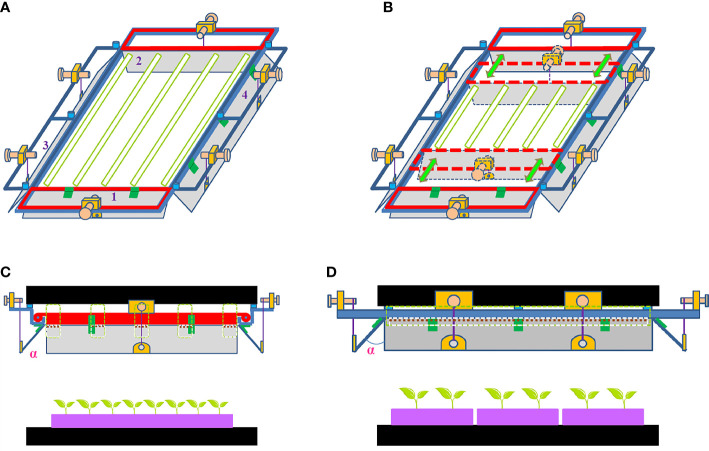
Adjustable lampshade-type reflector (ALR). **(A)** Three-dimensional schematic view of ALR with five LED tubes placed inside, illustrating its application in an indoor farm rack (IFR) lighting system. **(B)** Three-dimensional schematic view of ALR with five shorter LED tubes placed inside, illustrating its advantages for application in an IFR lighting system. **(C)** Front-width-side view of ALR installed at IFR. **(D)** Left-length-side view of ALR installed at IFR. α is the included angle of the reflective board. 1 and 2 in **(A)** stand for the first and second reflective boards coupled to the width sides of the main frame, while 3 and 4 in **(A)** represent the third and fourth reflective boards coupled to the length sides of the main frame.

The conceptual model of ALR was designed and custom-built according to the schematic diagrams shown in **Figure 1A**. Its aluminum-alloy-made main frame (120 cm × 60 cm) was composed of the opposing first and second width sides followed by the opposing first and second length sides. The first and second reflective boards, made of bright anodized aluminum, were pivotably coupled to the main frame at the first and second width sides of the main frame, respectively. The third and fourth reflective boards, made of the same material mentioned above, were also pivotably coupled to the main frame at the first and second length sides of the main frame, respectively, wherein the distance between the respective pivot axes of the first and second reflective boards was adjustable in order to regulate a reflective region of ALR based on the specification of light source applied (**Figure 1B**). All the reflective boards could be adjusted with an included angle that ranges from 0° to 90°. This allowed achieving optimal light distribution on an IFR cultivation shelf. The included angle (α) of the reflective board is defined as the intersection angle between the direction perpendicular to the ground and the reflective board as shown in **Figures 1C, D**.

### Simulation of the combined effects of included angle and reflective board width of ALR using TracePro software

In this novel reflector, the optimal combination of included angle and reflective board width under a certain distance between the light source and the germination tray surface is the key issue to be solved. However, it is a practically hard task to test all the included angles and various widths of the reflective boards because of the laborious works and exorbitant expenditures involved in order to seek an optimal combination for a well-performing reflector. Therefore, some optical software should be used for screening designs first.

**Figure 2:**
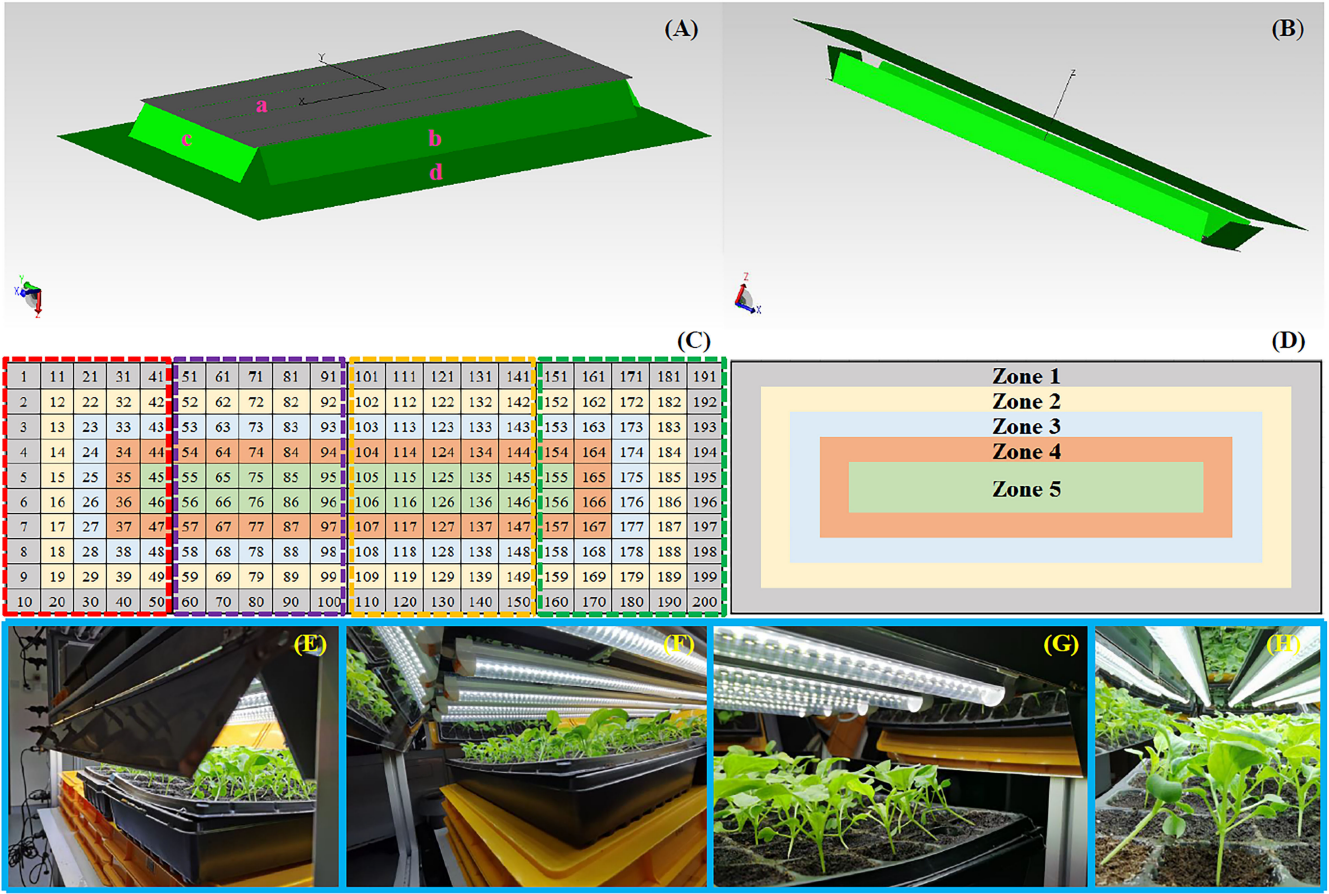
**(A)** Erected and **(B)** inverted schematic views of the three-dimensional simulative model of the adjustable lampshade-type reflector designed by the light simulative software TracePro. **(C)** Arrangement of four germination trays in the shelf of an indoor farm rack, marking the locations of a total of 200 cavities. **(D)** Definition of five zones on the four germination trays. Performance photos of the adjustable lampshade-type reflector observed from the viewing angles of **(E)** exterior, **(F)** short-side reflective board, **(G)** long-side reflective board, and **(H)** interior. The dashed rectangles with red, purple, earthy yellow, and green colors in **(C)** represent tray 1, 2, 3, and 4, respectively. In **(A)**, a is the lambertian diffuser board, b stands for the long-side simulative reflective board, c represents the short-side simulative reflective board, and d is the absorber board.

In this study, TracePro software (TracePro^®^ Expert—7.0.3 Release ACIS^®^, version 20.0.3, Lambda Research Corporation, USA), which includes a 3D CAD-based graphical user interface (Sun et al., 2014), was applied to simulate the light distribution maps across the IFR cultivation shelf with and without the adoption of an ALR under different combined conditions. This was to determine the optimal combination of the included angle and the reflective board width in a theoretical manner before a physical reflector was custom-built for performance validation. The parameter configurations in TracePro software required for the simulation are shown in **Table 1**, in which the specular reflectance of the simulated reflective boards was defined as 80%, the same as that of the bright anodized aluminum material employed in ALR as reflective boards (Powers and Dang, 1986). In this simulation, a five-LED-tube-constructed lighting group was designed and applied to the light source panel for the launching light rays to the cultivation shelf (**Figures 2A, B**).

**Table 1:** Parameter configuration of TracePro software for the simulation of the novel adjustable lampshade-type reflector.

Parameters	Configuration
Size of the lambertian diffuser board acting as the upper shelf of indoor farm racks (IFR) for installation of the simulated LED light tubes [length (mm) × width (mm)]	1,200 × 450
Size of the absorber board acting as the lower shelf of IFR [length (mm) × width (mm)]	1,200 × 600
Distance between the lambertian diffuser board and the absorber board (mm)	120
Size of the long-side reflective board [length (mm) × width (mm)]	1,130 × 100
Size of the short-side reflective board [length (mm) × width (mm)]	444 × 100
Parameters for the reflective board and LED light source information	
Scatter	ABg
Absorptance	20%
Specular reflectance	80%
Number of simulated LED light tubes	5
Distance between two simulated LED light tubes (mm)	110
Number of simulated LED chips in per tube	96
Distance between two simulated LED chips in per tube (mm)	11.7
Type of simulated LED chip	5,730
Size of simulated LED chip [length (mm) × width (mm) × height (mm)]	5.7 × 3.0 × 0.9
Parameters for simulated LED chip	
Emission type	Flux
Units	Photometric
Angular distribution	Lambertian
Flux (lumens)	27
Average wavelength (nm)	555
Simulated rays for per LED chip	2,000

Confirmation of the distance between the LED light source and the germination tray surface was also another key issue to be addressed, as a reduction of the distance could allow an IFR to accommodate more shelves, thus improving the IFR’s productivity. However, the canopy of choy sum shoots has a potential to touch the LED light sources during growth. Thus, an essential criterion is to prevent the shoot canopy from eventually touching the LED tubes before transplantation. As a result, the distance in this study was set as 12 cm by considering that the shoot canopy height of choy sum was normally approximately 8 to 9 cm on day 16 when the transplantation happened (Huang et al., 2021b). To coordinate with the distance, the width of the reflective board was thus pre-set as 10 cm.

**Figure 3:**
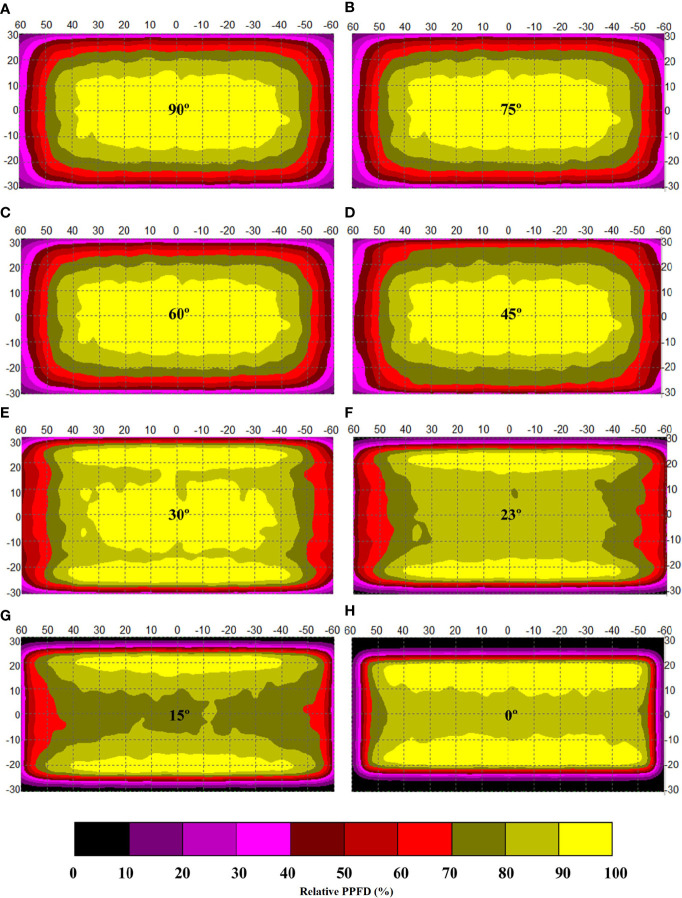
Sketchy screening of the appropriate included angles for the optimization of simulated light intensity distribution along an indoor farm rack shelf with the application of an adjustable lampshade-type reflector. Specifically, the screened included angles are **(A)** 90°, **(B)** 75°, **(C)** 60°, **(D)** 45°, **(E)** 30°, **(F)** 23°, **(G)** 15°, and **(H)** 0°, respectively. The width of the reflective board is set as 10 cm. The scale unit on the figures is centimeter. The numbers in the color bar indicate relative photosynthetic photon flux density values (%).

**Figure 4:**
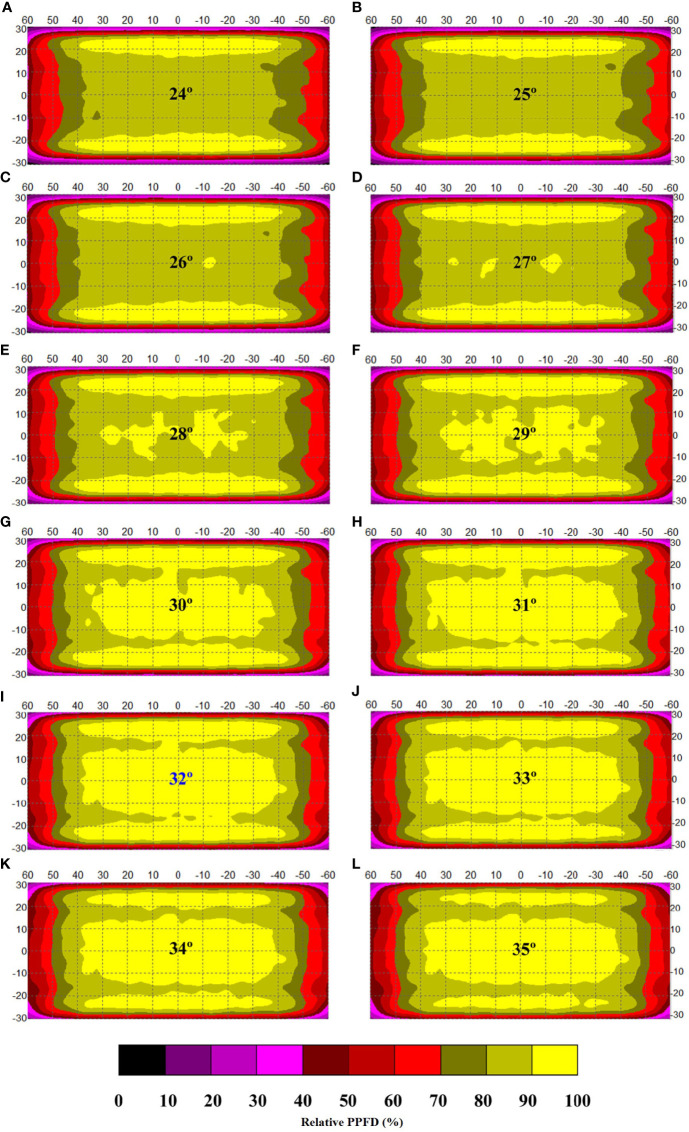
Fine-screening of the appropriate included angles for the optimization of simulated light intensity distribution along an indoor farm rack shelf with the application of an adjustable lampshade-type reflector. The screened included angles are **(A)** 24°, **(B)** 25°, **(C)** 26°, **(D)** 27°, **(E)** 28°, **(F)** 29°, **(G)** 30°, **(H)** 31°, **(I)** 32°, **(J)** 33°, **(K)** 34° and **(L)** 35°, respectively. The width of reflective board is set as 10 cm. The scale unit on the figure is centimeter. The numbers in the color bar indicate relative photosynthetic photon flux density values (%).

**Figure 5:**
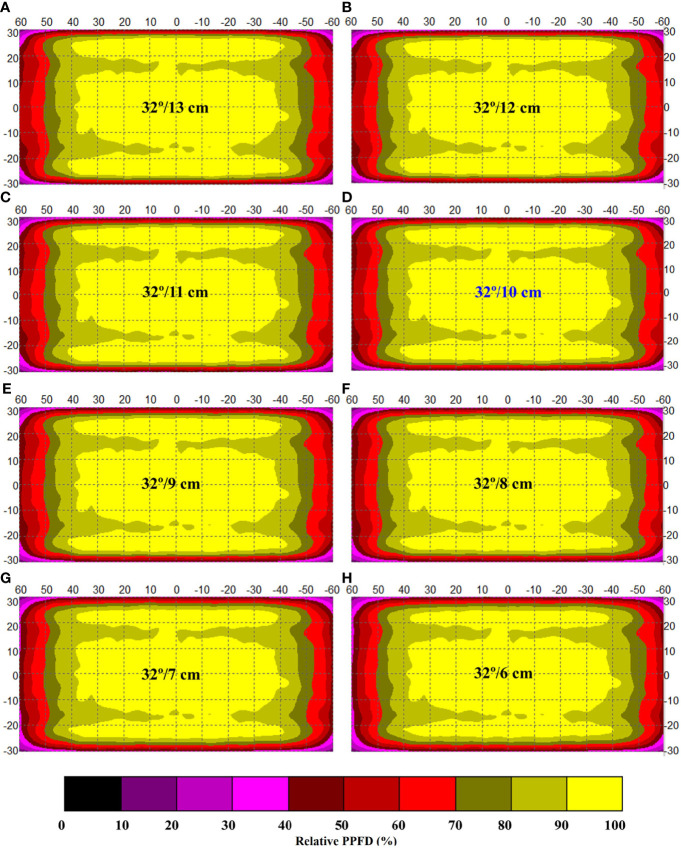
Screening of the appropriate width of the reflective board under the optimal included angle (32°) for the optimization of simulated light intensity distribution along an indoor farm rack shelf with the application of an adjustable lampshade-type reflector. The screened widths are **(A)** 13 cm, **(B)** 12 cm, **(C)** 11 cm, **(D)** 10 cm, **(E)** 9 cm, **(F)** 8 cm, **(G)** 7 cm, and **(H)** 6 cm, respectively. The scale unit on the figure is centimeter. The numbers in the color bar indicate relative photosynthetic photon flux density values (%).

The simulation procedure using TracePro was divided into three steps. The first step was aimed to confirm an optimal range of the included angle of the reflective board by sketchy screening. The selected angles were at a wide range including 90° (set as ALR-free control), 75°, 60°, 45°, 30°, 23°, 15°, and 0° (**Figure 3**). Referring to the sketchily screened range confirmed by step 1, the second step further fine-tuned the angle from 24° to 35° at 1° interval. This step confirmed the optimal included angle among these tested angles based on a 10-cm-width reflective board (**Figure 4**). The third step finally verified the optimal combination of the reflective board width and the included angle (*i*.*e*., the outcome of step 2) *via* testing boards of various widths ranging from 13 to 6 cm (**Figure 5**). In this testing, the available light distribution area (ALDA) was used as an index to describe the light distribution uniformity (LDU) of ALR, in which 10% and 15% standard deviations (SD) of PPFD were set as the thresholds for different LDU standards.

### Measurement of distribution maps of environmental factors under the selected included angles of ALR

**Figure 6:**
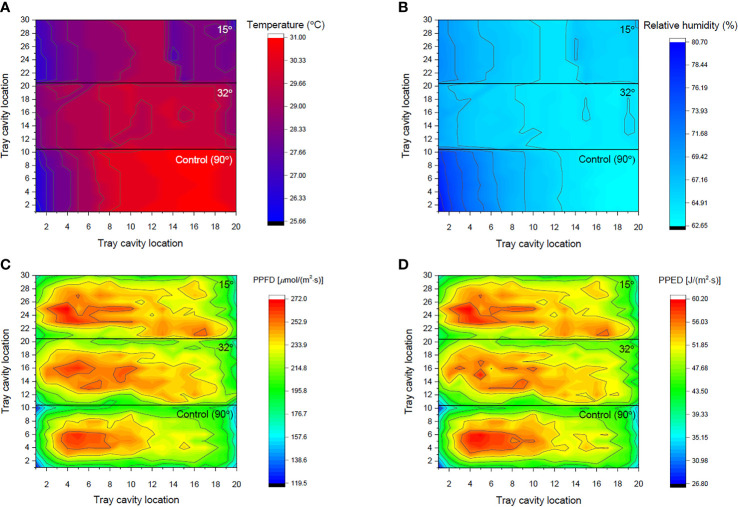
Heat maps of the measured **(A)** temperature, **(B)** relative humidity, **(C)** photosynthetic photon flux density on per cavity, and **(D)** photosynthetic photon energy density along the shelf of an indoor farm rack under different included angles of an adjustable lampshade-type reflector.

Four 50-cavity [each 5 cm (length) × 5 cm (width) × 4 cm (depth)] germination trays (Arianetech Pte Ltd., Singapore) were placed in a parallel manner on a shelf of the custom-built IFR (Arianetech Pte Ltd., Singapore). Five 22-W white LED tubes and ALR were then mounted at the lower part of the upper shelf (**Figures 1C, D**, **2E–H**). Various parameters related to the performance of the ALR, LED tubes, and trays were implemented in accordance with the simulated ones as shown in **Table 1**. **A** total of 200 cavities contained in the four trays were subsequently numbered and divided into five zones (*N*_zone 1_ = 56, *N*_zone 2_ = 48, *N*_zone 3_ = 40, *N*_zone 4_ = 32, *N*_zone 5_ = 24, *N*_total_ = 200, where *N* stands for the cavity number) (**Figures 2C, D**). Such a partition in zones was reasonable because the PPFD distribution in the center of a cultivation shelf is always higher than those gradually distant from the center, until the four shelf brims, in an annular and gradually decreasing manner due to the parallel arrangement of the five LED tubes. This phenomenon could be observed from both the simulation results (**Figure 3**) and the actual experimental outcome (**Figure 6C**). Generally speaking, the PPFD values within the same zone were similar or close to each other. The temperature, %RH, PPFD and PPED values at each tray cavity were measured in triplicate by using a light meter (ASENSETEK^®^ Lighting Passport, Taiwan). The experiments and measurements were conducted under three different conditions by adjusting the included angles of the four reflective boards to 15°, 32°, and 90° (control), respectively.

### Leafy vegetable cultivation

Choy sum seeds provided by Ban Lee Huat Seed Pte Ltd. (Singapore) were planted for us to conduct performance validation. Before sowing, each cavity of the four 50-cavity germination trays was filled with a standardized potting mix purchased from Jiffy^®^ (Jiffy Substrates, Toul, France). Each cavity was then sown with one seed. Subsequently, each tray was placed on the designated location of a cultivation shelf. To ensure that every cavity possessed a healthy seedling so as to make a fair comparison later, the fifth spare germination tray was sown simultaneously under the same light environment but was placed on another shelf. At 2 days after the sowing day (day 0), the non-germinated seeds in the first four trays were removed, and the corresponding cavities were replaced with healthy seedlings from the fifth tray. The photoperiod was set as 12:12 h of light/dark cycle daily. The seedlings were cultivated until the 16th day, which was the day set for harvest (Huang et al., 2021b). The experiments were performed in triplicate, respectively, under two conditions: (1) ALR-free control group (*i*.*e*., the included angle was set as 90°) and (2) ALR-implemented group where the included angle was 32°, which was confirmed by the simulation results as the optimal angle (**Figure 5D**). Each germination tray was irrigated with 3.0 L of water through sub-irrigation on the day of sowing (day 0) and subsequently watered with 1.0 L of tap water on days 4, 10, and 14, respectively.

### Determination of biomass and morphological parameters

The biomass and morphological parameters were determined by following the methods of Huang et al. (2021b). On harvest day (day 16), all seedlings, together with the soil, were carefully removed from the cavities of the germination trays. The roots with a soil portion were gently dipped into a beaker of clear tap water by sketchily rinsing off the soil while keeping the roots intact. The choy sum roots were subsequently fully cleaned using another beaker of clear tap water while care was taken to make sure that the roots were not broken during washing. Afterwards, the stretching state of the whole seedling or the flattened laminas from each seedling, as well as a ruler, were placed together and photographed using a smartphone (MIUI 8, China). Image analysis was conducted for the measurement of morphological parameters, including total leaf area (TLA), hypocotyl length (HL), hypocotyl diameter (HD), and root length (RL), by using ImageJ 1.51j8 software (National Institute of Health, Bethesda, MD, USA). The actual scales of all the items in the photos were referred to the ruler, and the photo scale was set accordingly. The length or diameter values were then determined by drawing a straight line along the items and were automatically calculated by the software, while the leaf areas were automatically measured by the software after drawing irregular and enclosed lines along the margin of the leaf.

After photographs had been taken, the fresh weight (FW) of each seedling was measured using a three-decimal-point electronic balance (Mettler Toledo ML303 Precision Balance, Greifensee, Switzerland). Each seedling was then cut into shoot and root parts by using a pair of scissors. The shoot and root parts were weighed separately and placed into a 50-ml Falcon tube. The tubes containing the respective seedlings from each cavity were frozen (DW-86L959BP, Haier, China) overnight at -80°C and then freeze-dried (Lyovapor™L-300, BUCHI, Switzerland) for 4 days. After lyophilization, the dry weight (DW) of each part of the seedling sample was determined. The shoot parts of the selected seedling samples were kept and utilized for the subsequent metabolite analyses of photosynthetic pigments, total phenolic content (TPC) and antioxidant capacity.

With reference to Tan et al. (2020), the harvest index (HI) and shoot/root ratio were determined using the following formula, respectively:


(1) $$HI=\frac{Choy\;sum\;shoot\;DW}{Whole\;choy\;sum\;seedling\;DW}$$

(2) $$Shoot/root\;ratio=\frac{Choy\;sum\;shoot\;DW}{Choy\;sum\;root\;DW}$$

### Determination of photosynthetic efficiency

With reference to Huang et al. (2016), the photosynthetic efficiency is defined as follows:


(3) $$PE(\%)=\frac{E_{B}}{E_{I}}\times 100\%$$

where *E*_B_ represents the free energy contained in the dry biomass of choy sum shoot (*i*.*e*., edible portion), and *E*_I_ stands for light energy in the spectrum of 380–780 nm containing the photosynthetically active radiation (PAR) range of 400–700 nm that was emitted by the white LED light tubes (Huang et al., 2021b). The light energy *E_I_* [J/(m^2^·s)] based on *I* [μmol/(m^2^·s)] is expressed as follows:


(4) $$E_{I}[J/(m^{2}\cdot s)]=h\times c\times A\times 10^{3}\times \sum_{\lambda =380}^{\lambda =780}\frac{I_{\lambda }}{\lambda }$$

where *h* is Planck constant (6.626×10^-34^ *J*·*s*), *c* represents the light ray velocity (2.998×10^8^ m/s), *λ* stands for photon wavelength (nm), *I_λ_* is light intensity [μmol/(m^2^·s)] under a certain wavelength from 380 to 780 nm, and *A* represents the Avogadro constant (6.022×10^23^/mol). *E*_B_ was calculated according to the following assumption: under a normal growth condition without stress, 100 g choy sum edible portion (containing 5.8 g DW) had 49 kJ of energy (Wills et al., 1984).

### Determination of pigments

For the measurement of pigments including chlorophyll *a* (chl*a*), chlorophyll *b* (chl*b*), total chlorophylls (Tchl), and total carotenoids (TC) in choy sum shoot, 30 shoot samples were selected from designated cavities to represent their respective zones, of which shoots from cavity numbers 5, 61, 70, 131, 140, and 195 were for zone 1, numbers 15, 62, 69, 132, 139, and 185 were for zone 2, numbers 25, 63, 68, 133, 138, and 175 were for zone 3, numbers 35, 64, 67, 134, 137, and 165 were for zone 4, and numbers 45, 65, 66, 135, 136, and 155 were for zone 5 (**Figure 2C**). As the cultivation experiments were conducted in triplicate, a total of 18 shoot samples were thus collected in each zone (*n* = 18). For pre-treatment, about 10 mg of weighed lyophilized choy sum shoots was quickly ground into powder in a pre-cooled mortar and pestle, which was covered with a black cloth to prevent the degradation of light-sensitive pigments. After that, 3, 3, and 4 ml of 80% acetone (HPLC grade, Sigma-Aldrich, St. Louis, MO, USA) in three consecutive steps, respectively, were added into the mortar to fully extract the powder. The powder together with the solvent was then transferred into a 15-ml Falcon tube; the tube was vortexed for 30 s and left to stand at 4°C for 24 h to completely release the pigments from the powder into the solvent. The mixtures were sonicated using a sonicator (Elmasonic S 60H, Singen, Germany) for 15 min and subsequently centrifuged at 3,500 × *g* for 15 min in a pre-cooled 4°C centrifuge tube (Thermo Scientific™ 75004533, Thermo Fisher Scientific, USA). After centrifugation, the supernatant was collected, and its absorbance was scanned from 200 to 780 nm to obtain the values at 663.6, 646.6, and 440.5 nm, respectively, by using a UV–vis spectrophotometer (UV1800, Shimadzu, Japan). The chl*a*, chl*b*, and TC contents were finally calculated using the following equations (mg per gram DW) (Huang et al., 2021a):


(5) $$Chla\;content\;(mg/gDW)=\frac{\left(12.25A_{663.6}-2.55A_{646.6})(mg/L\right)}{Dry\;weight\;(g\;DW/L)}$$

(6) $$Chlb\;content\;(mg/gDW)=\frac{\left(20.31A_{646.6}-4.91A_{663.6})(mg/L\right)}{Dry\;weight\;(gDW/L)}$$

(7) $$TC\;content\;(mg/gDW)=\frac{\left(4.69A_{440.5}-4.74A_{646.6}-1.96A_{663.6})(mg/L\right)}{Dry\;weight\;(gDW/L)}$$

### Sample preparation for antioxidant-related analyses

A total of 30 shoot samples were chosen to represent their respective zones. Shoots from cavity numbers 6, 71, 80, 121, 130, and 196 were for zone 1, numbers 16, 72, 79, 122, 129, and 186 were for zone 2, numbers 26, 73, 78, 123, 128, and 176 were for zone 3, numbers 36, 74, 77, 124, 127, and 166 were for zone 4, and numbers 46, 75, 76, 125, 126, and 156 were for zone 5, respectively (**Figure 2C**). With triplicate cultivation experiments, there were a total of 18 shoot samples for each zone (*n* = 18). For pre-treatment, 30 mg of freeze-dried choy sum shoot powder was added with 500 μl of acetone–water–acetic acid (70:29.5:0.5 v/v) and vortexed for 30 s to fully blend the mixture prior to sonication. The mixture was then sonicated in an ice bath for 15 min and centrifuged at 20,000 × *g* for 10 min at 4°C to collect the supernatants. The above-mentioned procedure was repeated twice, and the supernatants were pooled and then stored at -80°C prior to further analyses for TPC and antioxidant capacity (Huang et al., 2021a).

### Determination of total phenolic content

The TPC of choy sum shoot samples was determined using the Folin–Ciocalteu method with minor modifications (Ainsworth and Gillespie, 2007). Briefly, an aliquot of 100 µl of the above-mentioned shoot sample extract was blended with 200 µl of 10% Folin–Ciocalteu’s phenol reagent (Sigma-Aldrich, St. Louis, MO, USA), followed by 800 µl of 70 mM sodium carbonate solution, in a microtube and placed in the dark. After that, 200 µl of the sample, standard or blank, respectively, was transferred into microplate wells before the absorbance of each well at 765 nm was read using a microplate reader (Biotek SynergyMx, Vermont, USA). Different concentrations of gallic acid, ranging from 0.016 to 0.25 g/L, were used to establish a standard curve for calibration. The TPC of the choy sum shoots was determined, and the results were expressed as milligram gallic acid equivalents per gram dry weight of choy sum shoot (mg GAE/g DW).

### Determination of 2,2-diphenyl-1-picrylhydrazyl radical scavenging activity

In this study, 2,2-diphenyl-1-picrylhydrazyl (DPPH) radical scavenging activity assay was performed based on the microplate method developed by Bobo-García et al. (2015) with some modifications. Briefly, a total of 20 μl of the above-mentioned diluted choy sum shoot sample extract was mixed with 180 µl of 0.2 mM DPPH solution (Sigma-Aldrich, St. Louis, MO, USA) in methanol and then pipetted into the wells of a 96-well microplate. After incubating the mixtures in the dark at room temperature for 2 h, the absorbance was read at the wavelength of 515 nm on a microplate reader (Biotek SynergyMx, Vermont, USA). A standard curve of %DPPH quenched was established with the concentration of Trolox (Acros Organics, NJ, USA) ranging from 50 to 500 µM for calibration. The DPPH assay was carried out, and the results were expressed as micromole Trolox equivalents per gram dry weight of choy sum shoot (µmol TE/g DW).

The DPPH quenched (%) was calculated from Equation 8, where *A*_sample_ is the absorbance at 515 nm of 20 μl of the extract or standard with 180 μl DPPH solution after 2 h, *A*_blank_ is the absorbance at 515 nm of 200 μl methanol after 2 h, and *A*_control_ is the absorbance at 515 nm of 20 μl of water with 180 μl DPPH solution after 2 h.


(8) $$DPPH\;quenched(\%)=\left[1-\left(\frac{A_{sample}-A_{blank}}{A_{control}-A_{blank}}\right)\right]\times 100(\%)$$

### Statistical analysis

All the experiments, including cultivation, in this study were performed in triplicate (*n* = 3). The pigments, TPC, and DPPH of a total of 18 shoot samples for each zone (*n* = 18) were determined, respectively. Statistical analyses were performed using IBM Statistical Product and Service Solutions (SPSS version 17.0) software at a significant level of 0.05 (two-tailed). One-way analysis of variance (ANOVA) with Tukey’s multiple-comparison was used to evaluate the differences among all the zones or among all the included angles tested. Student’s *t*-test was employed to estimate the differences between two independent conditions (*e*.*g*., included angle of 90° as control and that of 32° as treatment). In the growth comparison experiments, the top 80% of choy sum seedlings, which had healthy development, were used for data analysis. The unhealthy and poorly developed choy sum seedlings were removed to minimize the negative effect of seed quality on the reliability of the experiment results.

## Results and discussion

### Optimal combination of included angle and reflective board width of ALR by simulation approach

As mentioned earlier, laborious works and exorbitant expenditures could be greatly saved through the application of software to simulate real situations. This demands that the simulation conditions must strictly follow the actual settings. Among the consistencies was the configuration of LED tubes in IFR, including the type, size, and number of LED chips. In addition, the consistent distances between any two chips, as well as between LED tubes and tray surface, *etc*., were also covered (**Table 1**). In this section, TracePro software was used to find the optimal combination of included angle and width of the ALR reflective board, based on which a physical reflector would be subsequently manufactured for *in situ* performance evaluation. As defined, the ALR included angles that ranged from 0° to 90°, among which an optimal angle existed to achieve the maximal reflection effect on retrieving stray LED light back to the IFR cultivation shelf. For a given LDU requirement (*e*.*g*., 10% or 15%), the larger the uniform area generated by an included angle on the cultivation shelf, the better the performance achieved by the angle.

To achieve the goal, a sketchy screening to find the approximately optimal included angle was firstly carried out by presetting the reflective board width as 10 cm, which was chosen based on the distance between the LED tube and the tray surface being 12 cm after considering the final canopy height of choy sum shoot achieved on day 16 (Huang et al., 2021b). As shown in **Figure 3** and **Table 2**, the included angles from 45° to 75° yielded minor variations in ALDA (within 10% SD of light intensity) which were almost the same as that of the control (3,406 cm^2^). However, ALDA under 30° of included angle was significantly increased to 4,694 cm^2^, which was remarkably higher than those under 23°, 15°, and 0°. The same tendency was also found under the LDU requirement of within 15% SD of light intensity, indicating that there should have been a peak value in a range approximately 30° that needed to be further investigated (**Figure 3** and **Table 2**). From the simulation results, ALDA under 30° of included angle was bigger than that under 0°, which is in line with the conclusion of Akiyama and Kozai (2016) that an inclined (board) reflector with appropriate angle (approximately 20°) was better than a vertical (board) reflector to improve PPFD across a cultivation shelf.

**Table 2:** Optimization of the included angles and widths of the four reflective boards of the novel adjustable lampshade-type reflector (based on a five-LED-tube panel).

Condition^a^	Included angle	Width (cm)	Highest light intensity [μmol/(m^2^·s)]	Within 10% SD of light intensity distribution			Within 15% SD of light intensity distribution		
				Area (cm^2^)	Percentage^b^ (%)	Average light intensity [μmol/(m^2^·s)]	Area (cm^2^)	Percentage (%)	Average light intensity [μmol/(m^2^·s)]
(1)	90°	10	253	3,406	47.3	228	4,369	60.7	215
(2)	75°	10	253	3,407	47.3	228	4,370	60.7	215
(3)	60°	10	253	3,409	47.4	228	4,371	60.7	215
(4)	45°	10	254	3,426	47.6	229	4,858	67.5	216
(5)	35°	10	253	4,763	66.2	228	5,476	76.1	215
(6)	34°	10	253	4,801	66.7	228	5,480	76.1	215
(7)	33°	10	253	4,817	66.9	228	5,481	76.1	215
(8)	**32°**	**10**	**253**	**4,830**	**67.1**	**228**	**5,496**	**76.3**	**215**
(9)	31°	10	256	4,808	66.8	230	5,481	76.1	218
(10)	30°	10	256	4,694	65.2	230	5,365	74.5	218
(11)	29°	10	263	4,598	63.9	237	5,349	74.3	224
(12)	28°	10	265	4,472	62.1	239	5,277	73.3	225
(13)	27°	10	269	4,347	60.4	242	5,210	72.4	229
(14)	26°	10	273	4,275	59.4	246	5,185	72.0	232
(15)	25°	10	276	4,173	58.0	248	5,142	71.4	235
(16)	24°	10	279	4,044	56.2	251	5,049	70.1	237
(17)	23°	10	280	3,938	54.7	252	4,997	69.4	238
(18)	15°	10	308	2,823	39.2	277	4,827	67.0	262
(19)	0°	10	320	4,108	57.1	288	4,439	61.7	272
(20)	32°	13	253	4,922	68.4	228	5,565	77.3	215
(21)	32°	12	253	4,895	68.0	228	5,533	76.9	215
(22)	32°	11	253	4,875	67.7	228	5,520	76.7	215
(23)	32°	9	253	4,785	66.5	228	5,440	75.6	215
(24)	32°	8	253	4,751	66.0	228	5,417	75.2	215
(25)	32°	7	253	4,697	65.2	228	5,385	74.8	215
(26)	32°	6	253	4,626	64.3	228	5,327	74.0	215

a Table background in yellow, green, and pink colors stands for the sketchy screening for the optimal range of the included angle, the fine screenings for the exact included angle, and the optimal combination of the included angle and the reflective board width, respectively.

b The available total shelf area for cultivation is 7,200 cm^2^.

SD, standard deviation. Bold fonts in the table meant the selected included angle and reflective board width under within 10% SD and 15% SD of light intensity distribution, respectively.

To locate the exactly optimal included angle in this scenario, a fine screening was further carried out. In this second screening, the included angles ranging from 23° to 35°, at 1° interval, were simulated to investigate the varying tendency of ALDA thus affected and to confirm the optimal included angle. With reference to **Figure 4** and **Table 2**, it was found that the peak ALDA value (67.1% of total shelf area), under the LDU requirement of within 10% SD of light intensity, fell on 32° of the included angle. Under the LDU requirement of within 15% SD of light intensity, the peak ALDA value (76.3% of total shelf area) was also found to be at 32°. Under both LDU requirements, an increasing trend was shown when the included angle increased from 23° to 32°, while a gradually decreasing trend was observed when the included angle further increased from 32° to 35° (**Table 2**). Compared with their respective reflector-free controls at 90°, the maximal ALDA values achieved by 32° under the LDU requirement of within 10% and 15% SD of light intensity were enhanced by 41.8% and 25.8%, respectively. Thus, 32° was finally selected as the optimal included angle to achieve the maximal ALDA value across a cultivation shelf, compared with all the other simulated angles with the same 10-cm width of the ALR reflective board (**Figure 4** and **Table 2**).

The last and the most important step was to figure out the best combination of included angle and reflective board width that can provide a better cultivation environment for plants compared with the reflector-free control. After the above-mentioned confirmation of the optimal included angle (*i*.*e*., 32°), the optimization of the ALR reflective board width under this angle was eventually carried out by testing the variation of ALDA under various widths of the reflective board, ranging from 6 to 13 cm at 1-cm interval. Based on **Figure 5** and **Table 2**, it was obvious that ALDA (no matter within 10% or 15% SD of light intensity) was the largest, compared with all the other tested widths, when the board width was 13 cm. Furthermore, a decreasing tendency of area was found with the reduction of width, indicating that a larger width may help to reflect more of the stray light rays back to the IFR cultivation shelf under the same included angle. This is in agreement with the results from a previous reflector-related study showing that the average PPFD across the shelf under 15 cm of the side reflector width is 10% and 25% more than those under the 10- and 0-cm (no-reflector control) ones, respectively (Akiyama and Kozai, 2016).

Besides ALDA, two other issues should also be addressed when determining an optimal ALR reflective board width. Firstly, the ventilation issue has to be considered to match the preset distance between the LED tubes and the tray surface. In this study, the distance was 12 cm, which was to guarantee that the canopy height of choy sum shoot could grow up to approximately 8 to 9 cm on day 16 (Huang et al., 2021b) and the canopy would receive as much light as possible without touching the LED tubes. Meanwhile, the IFR productivity could be significantly improved under such a distance. This is because the shorter shelf height achieved of 19 cm (noting the 5-cm height of the germination tray and the 2-cm height of the LED tubes) can accommodate more shelves in the IFR compared with 27 cm of the original IFR shelf height currently applied in our indoor plant factory. Therefore, the ALR reflective board width is suggested not to exceed 12 cm, considering that efficient ventilation should be maintained across the cultivation shelf for the entire seedling growth period. Another factor that needs to be considered is the material cost to manufacture the ALR reflective boards. The larger the width of an ALR reflective board that is applied, the higher the board would cost. Thus, 10 cm was chosen as the optimal width to save on the board material and its cost because ALDA under 10 cm was close to that under 11 cm (**Table 2**). From this simulation study, the combination of 32° of included angle and 10 cm of reflective board width was therefore selected as optimal, which acted as the basis for manufacturing a physical reflector to facilitate an *in situ* performance evaluation as described below.

### Comparison between the distribution of environmental factors on the cultivation shelf with and without ALR application

**Figure 7:**
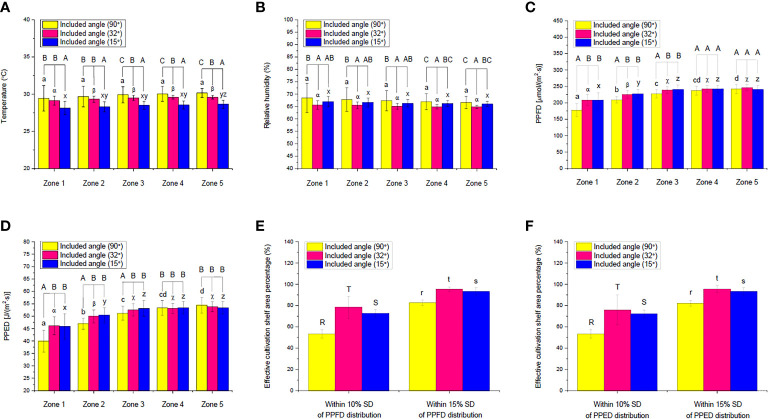
Comparisons of the measured **(A)** temperature, **(B)** relative humidity, **(C)** photosynthetic photon flux density (PPFD) and **(D)** photosynthetic photon energy density (PPED) in different shelf zones of an indoor farm rack under different included angles of an adjustable lampshade-type reflector as well as effective cultivation shelf area percentages within different SD of PPFD distribution under **(E)** PPFD-based and **(F)** PPED-based conditions. Different uppercase letters (A, B, and C) indicate significant differences among different included angles in the respective zones; different lowercase letters (a, b, c, d, x, y, and z) and Greek alphabets (α, β, and χ) indicate significant differences among all the zones under the respective included angles; different uppercase letters (R, S, and T) and lowercase letters (r, s, and t) indicate significant differences among the different included angles within 10% and 15% SD of the PPFD/PPED distribution along the whole shelf, respectively (*N*_zone1_ = 56, *N*_zone2_ = 48, *N*_zone3_ = 40, *N*_zone4_ = 32, *N*_zone5 =_ 24, *N*_total_ = 200, one-way ANOVA; Tukey multiple-comparison; *p*< 0.05).

After the optimal combination of included angle and reflector board width was determined, a custom-built ALR was manufactured (**Figures 2E–H**) to further investigate the actual performance and efficiency of this novel reflector. The first evaluation step was to compare the distribution of key environmental factors such as temperature, %RH, PPFD, and PPED on each cavity along a cultivation shelf between with and without the ALR. The acquired data could help us to check the distribution uniformity of these factors under selected included angles. A more uniform environment may facilitate the development of a more uniform morphology of seedlings and improve the total biomass accumulation. In this study, the included angles of 15°, 32° (the optimal simulated angle), and 90° (reflector-free control) were selected (**Figures 6**, **7**).

As shown in **Figure 6A**, 32° of included angle could achieve the most uniform temperature distribution along a cultivation shelf, compared with those under 15° and 90° (the control). Under the control, the temperature range of the 200 cavities was 25.7–31.0°C, which indicates that the distribution was inhomogeneous. However, the temperature difference along the cultivation shelf was significantly smaller under the ALR. The temperature range became 28.0–30.0°C and 26.7–29.3°C when the included angle was 32° and 15°, respectively. Furthermore, from **Figure 7A**, the 90° control group displayed the biggest SD, resulting in an insignificant difference among their five zones, while the 32° and 15° groups demonstrated smaller SD values, leading to significant differences between zone 1 and the other zones under 32° as well as between zones 1 and 5 under 15°. This indicates that the ALR application could markedly minimize the temperature range/distribution difference along a cultivation shelf.

Among all the zones, the temperature values in zones 3, 4, and 5 of the control group were remarkably higher than those under the ALR of the 32° and 15° groups, respectively (**Figure 7A**). This could be due to the property of reflective boards made of bright anodized aluminum that absorbs part of the heat from the surroundings, as the spectral absorptivity of this material to far-red light (701–780 nm) may range from 35% to 60% (Moghadam et al., 2013). In addition, the temperature under the ALR of 32° included angle group was significantly higher than that under the 15° one across all the zones. This could be due to the fact that a smaller included angle (*i*.*e*., 15°) generated more enclosed room for the board to efficiently absorb heat, resulting in a significant reduction of temperature across the cultivation shelf under the ALR of 15° included angle (**Figure 7A**).

Another interesting phenomenon observed was that 32° of included angle was superior to 15° in terms of uniform temperature distribution along the shelf (**Figure 6A**). One reason might be that an appropriate included angle like 32° could help to reflect not only visible light but also far-red light to a wider cultivation area owing to the reflectance property of the bright anodized aluminum (Li et al., 2021). However, an included angle like 15° mainly reflected far-red light onto the shelf center, thus lowering the average temperature values in all the zones and generating bigger SD (**Figure 6A**).

The experimental results showed that the %RH distribution across a shelf was inversely related with that of the temperature (**Figures 6A, B**). The absolute amount of water vapor or absolute humidity within the atmosphere of a shelf was relatively constant (Bencloski, 1982), regardless if an ALR was used. Similar to the temperature distribution, the %RH values of the control group were in the range of 62.7%–80.7%, which was larger than those under the ALR of the 32° and 15° groups (63.7%–69.3% and 64.3%–71.0%, respectively). Thus, the ALR also minimized the %RH distribution difference along the cultivation shelf (**Figure 7B**). In the control group, the %RH SD was the highest in zone 1 and then gradually decreased until zone 5. In addition, the significant %RH differences among the control and the 32° and 15° groups in different zones were mainly caused by the bigger SD in the control group. Although there was no statistically significant difference among the five zones under the three respective groups, the %RH distribution under the 32° included angle group was the most even (**Figure 6B**). Therefore, the ALR of the 32° included angle was also confirmed to be able to improve the uniformity of %RH distribution along a cultivation shelf.

Equally important mentioning is that the PPFD and PPED on each cavity were the other two essential factors that are closely related to the growth and quality of a plant. PAR could be especially fully captured and utilized by the plant for photosynthesis (Huang et al., 2020). In this study, comparisons of the two factors and the effective cultivation shelf area percentages based on them (within 10% and 15% SD) were made (**Figures 6C, D**, **7C–F**). The average PPFD and PPED under the control and ALR conditions were both progressively and significantly increased from zones 1 to 5. This is expected because light distributions around the center of a cultivation shelf are always higher than those outside it until the brim in a gradually decreasing manner. However, the values of these two factors under the ALR conditions from zones 1 to 3 were remarkably higher than those under the control. This indicates that implementing an ALR could dramatically improve the PPFD and PPED of those cavities that were located particularly at the brim and off-center areas of the shelf compared with the control. An improvement to enhance the brim PPFD was also observed in a plant lighting system equipped with a LED panel surrounded by four plates of 10 cm in width (Yano and Fujiwara, 2012). Actually, that plate-equipped LED panel system performed in a similar way to the condition of 0° included angle in our study, under which heat dissipation and ventilation would be non-negligible issues as explained earlier. In general, the above-mentioned results strongly support the objective of this invented ALR, *i*.*e*., to retrieve stray light energy towards plant growth on the shelf. Furthermore, it was found that the statistical differences under the ALR application from zones 1 to 5 were markedly decreased compared with the control, substantiating the efficiency of ALR (**Figures 7C,D**).

Compared with the levels of PPFD and PPED on each cavity under the reflector-free control [which were 119.6 (min) to 265.8 (max) μmol/(m^2^·s) and 26.9 (min) to 59.8 (max) J/(m^2^·s), respectively], the ALR application under 32° produced 164.4 to 264.7 μmol/(m^2^·s) and 29.2 to 56.9 J/(m^2^·s), respectively, and that under 15° yielded 158.5 to 271.7 μmol/(m^2^·s) and 35.0 to 60.2 J/(m^2^·s), respectively. The ALR applications tremendously enhanced the PPFD and PPED on each cavity at the brim of a cultivation shelf and significantly decreased the difference within the shelf. Upon further examination of the heat maps (**Figures 6C, D**), the ALR of 32° and 15° included angles were found to retrieve back more PPFD and PPED onto the shelf and distribute them more evenly along the shelf compared with the control. Thus, the included angle of 32° was superior to 15° by aggregating less PPFD/PPED on the specific cavities of the trays, facilitating a more uniform development of the seedlings on the cultivation shelf (**Figures 6C, D**). The included angle of 32° was thus confirmed again as the best ALR included angle among all the angles tested.

Comparing the effective cultivation shelf area percentages (within 10% and 15% SD of PPFD/PPED) under all the included angles tested, the effective areas under 32° were significantly higher than those under 15° and the control, while those under 15° were also markedly higher than the control. Compared with the control, the included angle of 32° increased the effective area by up to 47% and 15% based on PPFD for 10% and 15% SD, respectively, as well as by 42% and 16% based on PPED for 10% and 15% SD, respectively (**Figures 7E, F**). An appropriate ALR application can thus effectively increase the effective cultivation shelf area.

### Effect of ALR application on the growth and morphology of choy sum seedling

After the effectiveness of ALR on improving the key environmental conditions on a cultivation shelf was confirmed, validating the performance of ALR in real plant cultivation on the shelf would be essential to eventually prove that this reflector offers benefits to an indoor plant factory. As such, a popular leafy vegetable rich in bioactive metabolites and widely cultivated in Asia, choy sum, was chosen as the experimental plant (Liang et al., 2018; Huang et al., 2021a).

**Figure 8:**
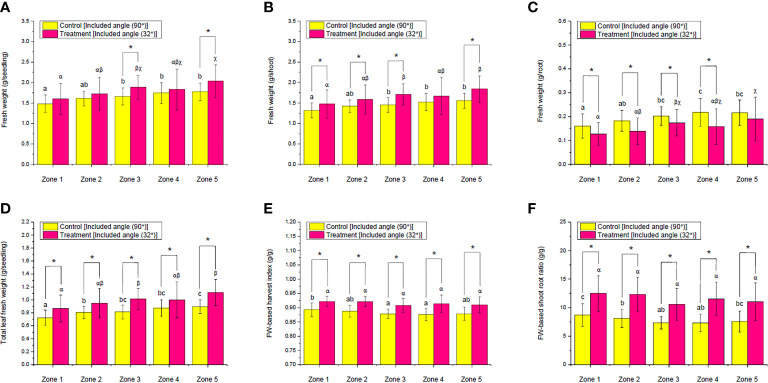
Comparison of the fresh weight (FW) of **(A)** seedling, **(B)** shoot, **(C)** root, and **(D)** total leaf as well as **(E)** FW-based harvest index and **(F)** FW-based shoot–root ratio of choy sum at seedling stage on day 16 grown in an indoor farm rack with and without the application of an adjustable lampshade-type reflector (ALR). The asterisk (*) indicates significant differences found between the control and treatment (Student’s *t*-test; *p*< 0.05), while different Greek alphabets (α, β, and χ) and the lowercase letters (a, b, and c) indicate significant differences among all the zones with and without the application of ALR (*N*_zone1_ = 45, *N*_zone2_ = 38, *N*_zone3_ = 32, *N*_zone4_ = 26, *N*_zone5 =_ 19, one-way ANOVA; Tukey multiple-comparison; *p*< 0.05).

In this case, the influences of ALR on the FW of choy sum seedling, shoot, root, and total leaf, as well as the FW-based harvest index and shoot/root ratio, were firstly determined and compared between the control and ALR with the optimal included angle of 32° (**Figure 8**). Compared with the control, the seedling FW in zones 3 and 5 under the ALR of 32° included angle was significantly increased by up to 13% and 14%, respectively (**Figure 8A**). In addition, the shoot FW in zones 1, 2, 3, and 5 were also markedly enhanced by 12%, 11%, 17%, and 18%, respectively (**Figure 8B**). In addition, the total leaf FW (**Figure 8D**), FW-based harvest index (**Figure 8E**), and shoot/root ratio (**Figure 8F**) were remarkably increased across all the zones. In contrast, the root FW in zones 1, 2, 3, and 4 was significantly decreased (**Figure 8C**). It is worth to note that there was a significantly increasing trend from zone 1 to 5 in terms of FW of seedling, shoot, root, and total leaf under both the control and ALR conditions. This was concurrent with the gradual increases of PPFD distribution from zones 1 to 5 (**Figure 7C**), as the biomass accumulation of choy sum seedling was always positively related to light intensity below its light saturation point (Huang et al., 2021b). Nevertheless, the FW-based harvest index and shoot/root ratio under ALR did not show a significant difference among all the zones, although fluctuations across the zones were found under the control (**Figures 8E, F**). These results supported that implementing an ALR could increase the above-ground FW biomass of choy sum seedling along an IFR cultivation shelf as well as the uniformity of the biomass across the shelf.

**Figure 9:**
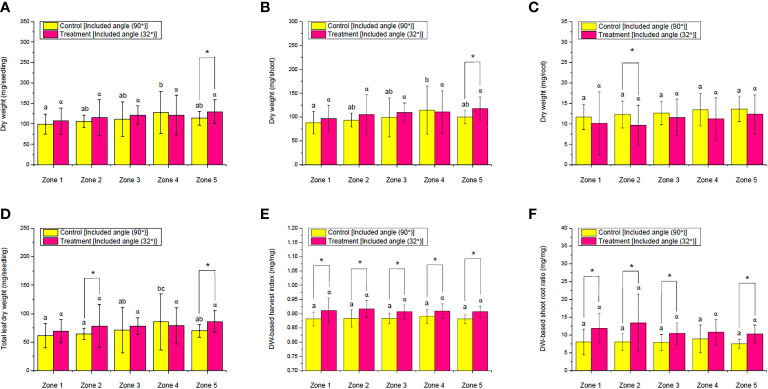
Comparison of the dry weight (DW) of **(A)** seedling, **(B)** shoot, **(C)** root, and **(D)** total leaf as well as **(E)** DW-based harvest index and **(F)** DW-based shoot–root ratio of choy sum at seedling stage on day 16 grown in an indoor farm rack with and without the application of an adjustable lampshade-type reflector (ALR). The asterisk (*) indicates significant differences found between the control and treatment (Student’s *t*-test; *p*< 0.05), the lone Greek alphabet (α) indicates that there is no significant difference found among all the zones with the application of ALR, and the lowercase letters (a, b, and c) indicate significant differences among all the zones without the application of ALR (*N*_zone1_ = 45, *N*_zone2_ = 38, *N*_zone3_ = 32, *N*_zone4_ = 26, *N*_zone5 =_ 19, one-way ANOVA; Tukey multiple-comparison; *p*< 0.05).

The influences of ALR on the DW of choy sum seedling, shoot, root, and total leaf, as well as on the DW-based harvest index and shoot/root ratio, were likewise subsequently evaluated (**Figure 9**). Compared with the control, the DW of an average seedling and shoot in zone 5 was significantly increased by up to 14% and 18%, respectively. However, only root DW in zone 2 was remarkably decreased instead. Except for that, there was no significant difference found in other zones between the control and the ALR of 32° included angle (**Figures 9A–C**). For total leaf DW, the implemented ALR resulted in a significant increase by up to 22% and 24% in zones 2 and 5, respectively, compared with the control (**Figure 9D**). Particularly, a significant increase in the DW-based harvest index among all the zones was observed due to the ALR implementation (**Figure 9E**). Moreover, similar outcomes among the zones were found in the DW-based shoot/root ratio, except for zone 4 (**Figure 9F**). When comparing the DW of seedling, shoot, root, and total leaf among all the zones within the same group, no significant difference in the DW of all parts of choy sum was observed in the ALR group. In the control group, however, the DW values of these parts in choy sum were fluctuating across the zones (**Figures 9A–D**). These findings again indicated that the ALR implementation could increase the developmental uniformity of choy sum seedlings among the different zones of an IFR shelf.

**Figure 10:**
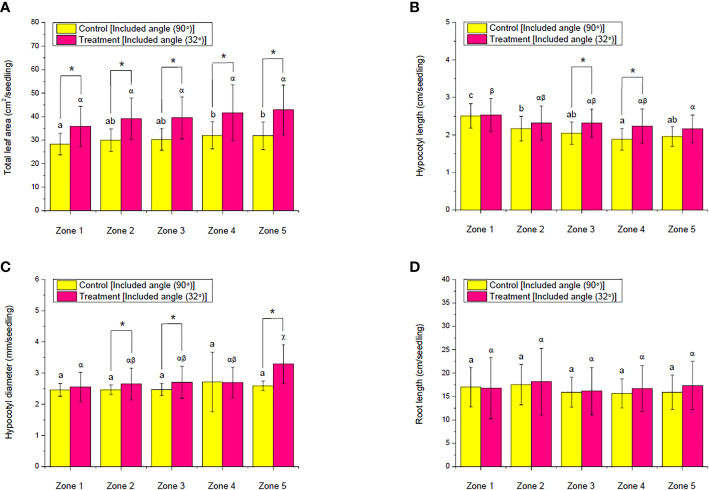
Comparison of the **(A)** total leaf area, **(B)** hypocotyl length, **(C)** hypocotyl diameter, and **(D)** root length of choy sum at seedling stage on day 16 grown in an indoor farm rack with and without the application of an adjustable lampshade-type reflector (ALR). The asterisks (*) indicate significant differences found between the control and treatment (Student’s *t*-test; *p*< 0.05), while different Greek alphabets (α, β, and χ) and the lowercase letters (a, b, and c) indicate significant differences among all the zones with and without the application of ALR (*N*_zone1_ = 45, *N*_zone2_ = 38, *N*_zone3_ = 32, *N*_zone4_ = 26, *N*_zone5 =_ 19, one-way ANOVA; Tukey multiple-comparison; *p*< 0.05).

The morphological response of choy sum seedlings to the implemented ALR showed a similar outcome. The average TLA of each seedling in all the zones increased by up to 34%, compared with the control (**Figure 10A**), which was primarily because the implemented ALR increased the PPFD and PPED on the cultivation shelf by reflecting stray light back (**Figures 7C–F**), provided that the light intensity was below choy sum’s light saturation point (Huang et al., 2021b). The ALR group also did not have any significant difference among all the zones, while the control group showed a gradual and remarkable increase, indicating that the implemented ALR helped to achieve a more uniform leaf development across all the zones (**Figure 10A**). In addition, under both the control and ALR conditions, the shoot HL from zones 1 to 5 were gradually and significantly shortened, pointing towards the fact that the PPFD and PPED at the shelf center were significantly higher than those at the brim, as choy sum HL was found to be negatively related to light intensity in our earlier studies (Huang et al., 2021b). However, the shortening of HL in the control group was more significant than that of the ALR group, and the control group HL in some zones, such as zones 3 and 4, were markedly shorter than those in the ALR group. This revealed that the more uniform HL was achieved by the implemented ALR compared with the control (**Figure 10B**).

Looking into the shoot HD, the control group did not show any significant difference across all the zones, while the ALR group had a gradual increase from zones 1 to 5, which also led to significantly higher HD in some zones (*i*.*e*., 2, 3, and 5) than the control group (**Figure 10C**). The positive correlation between light intensity and choy sum hypocotyl diameter could explain the current HD results (Huang et al., 2021b). Regarding RL, no significant difference was found either between the control and ALR conditions or across the zones under the same condition (**Figure 10D**). In short, the morphological responses of choy sum seedling to the implemented ALR indicated that ALR could help to facilitate a more uniform morphological development of choy sum seedling along a cultivation shelf.

**Figure 11:**
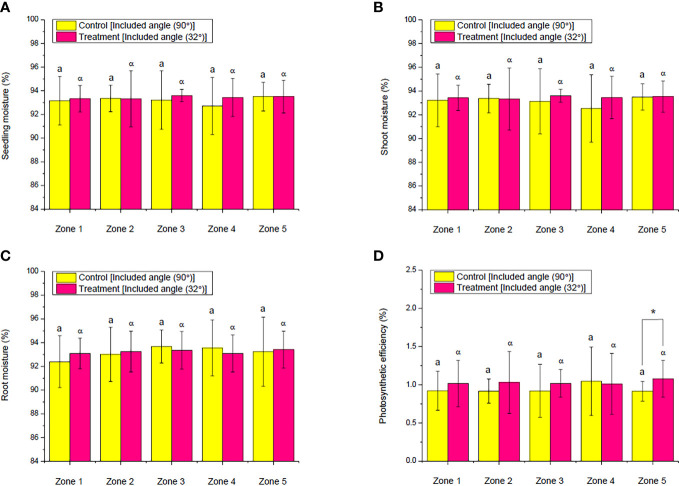
Comparison of the moisture of **(A)** seedling, **(B)** shoot, and **(C)** root as well as **(D)** photosynthetic efficiency of choy sum at seedling stage on day 16 grown in an indoor farm rack with and without the application of an adjustable lampshade-type reflector (ALR). The asterisks (*) indicate significant differences found between the control and treatment (Student’s *t*-test; *p*< 0.05), while the lone Greek alphabet (α) and the lowercase letter **(a)** indicate that there are no significant differences found among all the zones with and without the application of ALR (*N*_zone1_ = 45, *N*_zone2_ = 38, *N*_zone3_ = 32, *N*_zone4_ = 26, *N*_zone5 =_ 19, one-way ANOVA; Tukey multiple-comparison; *p*< 0.05).

Observing the moisture variations of seedling, shoot, and root between the control and ALR groups and across the zones within the same group, there was not any significant difference found (**Figures 11A–C**). Furthermore, except PE in zone 5 under the ALR condition, which was remarkably higher than that under the control, PE in other zones between the control and ALR groups as well as across zones 1 to 4 within the same group did not demonstrate any significant difference (**Figure 11D**). The PE as presented here is a vital index that represents a plant’s capability to capture and convert light energy into its chemical potential energy (Huang et al., 2016; Huang et al., 2021b).

### Effect of ALR application on the pigments of choy sum shoot

**Figure 12:**
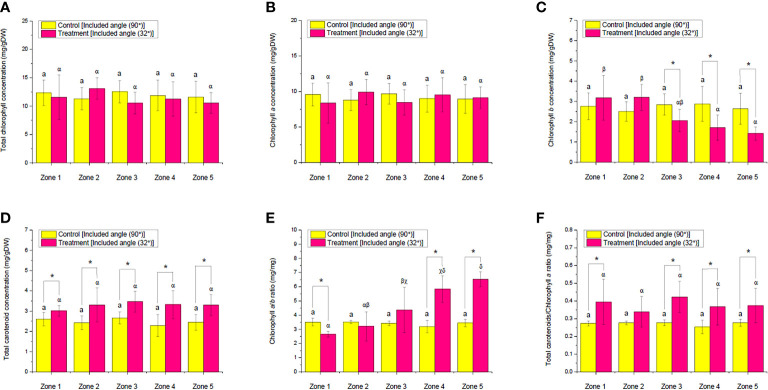
Comparison of the concentration of **(A)** total chlorophylls, **(B)** chlorophyll *a*, **(C)** chlorophyll *b* and, **(D)** total carotenoids as well as **(E)** chlorophyll *a*/*b* ratio and **(F)** total carotenoids/chlorophyll *a* ratio of choy sum shoots at seedling stage on day 16 grown in an indoor farm rack with and without the application of an adjustable lampshade-type reflector (ALR). The asterisks (*) indicate significant differences found between the control and treatment (Student’s *t*-test; *p*< 0.05), the lone lowercase letter (a) indicates that there is no significant difference found among all the zones without the application of ALR, and different Greek alphabets (α, β, χ, and δ) indicate significant differences found among all the zones with the application of ALR (*N*_zone1_ = 6, *N*_zone2_ = 6, *N*_zone3_ = 6, *N*_zone4_ = 6, *N*_zone5_ = 6, one-way ANOVA; Tukey multiple-comparison; *p*< 0.05).

As for the pigment responses of choy sum shoots to ALR application, the levels of chl*a*, chl*b*, and Tchl, as well as TC, under the control and ALR conditions were investigated (**Figure 12**). There was no significant difference in the levels of chl*a* and Tchl between the control and ALR groups and also across the zones within the same group. The level of chl*b* in the control group was statistically the same across all the zones (**Figures 12A–C**). This revealed that the presence of ALR did not affect the biosynthesis of chlorophyll pigments significantly. However, the level of chl*b* in the ALR group remarkably decreased from zones 1 to 5 (**Figure 12C**). As chl*b* is normally negatively correlated to light intensity (Li et al., 2012; Huang et al., 2021a), the reduction of chl*b* level from zones 3 to 5 was consistent in such a way that the light intensities across these areas were significantly enhanced through the ALR application (**Figures 7C**, **12C**). On the contrary, the levels of TC in the control and ALR groups did not show any significant difference across the zones within the same group. Nevertheless, significant differences were found between the control and ALR groups. The levels of TC in all five zones of the ALR group were all markedly higher than their counterparts in the control group by up to 45% (**Figure 12D**). It was well documented in the literature that carotenoids might possess a light-harvesting property and function in the energy transfer process through capturing purple to blue light photons and delivering energy to chl*a* molecules for photosynthesis (Nurachman et al., 2015; Huang et al., 2017). Therefore, the enhanced TC level in the ALR group could be due to the improved light environment on the cultivation shelf, such as higher PPFD and PPED and more uniform light distribution, thus inducing carotenoid biosynthesis to seize more light energy to support plant growth (**Figures 6C, D**, **7C, D**, **12D**). As carotenoids have been shown to benefit human wellness by acting as antioxidants, anti-inflammatory agents, and anti-cancer compounds (Huang et al., 2017), the implementation of ALR could thus be proposed as an effective approach to improve the nutritional quality of choy sum shoot while using the same amount of electricity as the control.

To further observe two essential parameters—chl*a*/chl*b* and TC/chl*a* ratios—that are closely related to photosynthesis and light stress (Filella et al., 2009; Huang et al., 2020; Huang and Cheung, 2021), the chl*a*/*b* ratio in the ALR group was found to increase sharply from zones 1 (which had relatively low PPFD) to 5 (which had relatively high PPFD) compared with the control group that did not show any significant difference across the five zones. However, the chl*a*/chl*b* ratio at the shelf brim (*i*.*e*., zone 1) in the ALR group was remarkably lower than that in the control group, while the ratio at the shelf center (zones 4 and 5) was markedly higher than that of the control (**Figure 12E**). It was clear that the significant increase of chl*a*/chl*b* ratio from zones 1 to 5 was corresponding to the reduction of the chl*b* level (**Figure 12C**). The upregulation of this parameter could help to minimize the size of auxiliary light-harvesting chlorophyll antenna for maximizing PE (**Figure 11D**) (Melis, 2009; Huang and Cheung, 2021).

**Figure 13:**
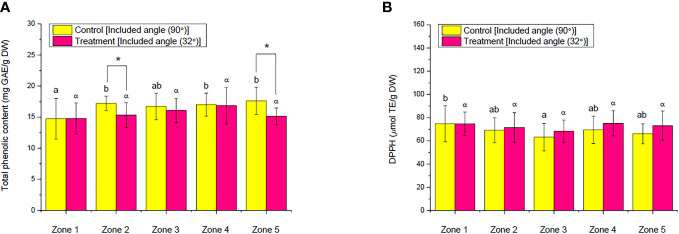
Comparison of the **(A)** total phenolic content and **(B)** 2,2-diphenyl-1-picrylhydrazyl of choy sum shoots at seedling stage on day 16 grown in an indoor farm rack with and without the application of an adjustable lampshade-type reflector (ALR). Different Greek alphabets (α and β) and the lowercase letters (a, b, and c) indicate significant differences among all the zones with and without the application of ALR (*N*_zone1_ = 6, *N*_zone2_ = 6, *N*_zone3_ = 6, *N*_zone4_ = 6, *N*_zone5_ = 6, one-way ANOVA; Tukey multiple-comparison; *p*< 0.05).

On the other hand, the TC/chl*a* ratio of the ALR group was found to be significantly higher than its counterpart in the control group across all the zones except zone 2. Nevertheless, there was no significant difference across the zones within the same group (**Figure 12F**). Although the TC/chl*a* ratio is usually recognized as an indicator of light stress because one of carotenoid’s role is to scavenge reactive oxygen species induced by light stress (Filella et al., 2009), the current results seemed to support the other role of carotenoid, as a light-harvesting tool, that was described earlier. Because the light-harvesting complexes of photosystem II (LHC II) in vegetables are usually more than one type and the carotenoid compositions in the respective LHC II are commonly different (Phillip and Young, 1995), the wane and wax of LHC II under different plant growth conditions have potential to upregulate the TC proportion of LHC II. Thus, it was possible that a stable chl*a* level was maintained, whereas a higher TC level in the vegetable was achieved when the environment was changed (**Figures 12B, D, F**). However, future studies should look into the profile of carotenoid in our choy sum case for clarification. It is worth noting that the implementation of ALR in this study was not intended to generate a light stress environment for plants, which could be further proved by the TPC and DPPH results (**Figure 13**) that are to be discussed in the following section. Overall, the TC/chl*a* ratio in the ALR group was significantly enhanced by up to 52.5% compared with that of the control group (**Figure 12F**).

### Effect of ALR application on total phenolic contents and the antioxidant capacity of choy sum shoot

Phenolic compounds are the essential secondary metabolites found in plants, which function in the protection of plants against biotic and abiotic stresses (Wong et al., 2020). Thus, they could be utilized as indicators to check whether the plants are under a stressed light environment or not. In this work, the TPC and DPPH assays were conducted to compare the level of antioxidant-related metabolites and the antioxidant activities of choy sum shoots cultivated on an IFR shelf with and without the implementation of an ALR. As shown in **Figures 13A, B**, the implementation of ALR did not significantly influence the TPC and DPPH values of choy sum shoots across all the zones, indicating that the PPFD and PPED distribution along the cultivation shelf was more uniform by using an ALR compared with the reflector-free control. The TPC and DPPH values in the control group were fluctuating across the zones due to the fluctuating light distribution in the absence of ALR (**Figures 6C, D**). Owing to the uneven distribution of PPFD and PPED under the control, the levels of phenolics in some zones, such as zones 2 and 5, were markedly higher than those in the ALR group (**Figure 13A**). However, this led to the heterogeneity of choy sum seedling quality along the shelf in the absence of ALR.

## Conclusion

The implementation of an ALR with optimized angle, distance, *etc*., can minimize the distribution differences of temperature and relative humidity along an IFR cultivation shelf. It can also enhance the PPFD and PPED distribution along the cultivation shelf. Such improvements could significantly improve the growth and morphological traits, including the nutritional quality of choy sum seedlings in indoor farm practice. In short, the ALR designed in this study is suitable and efficient to be applied in indoor plant factories for leafy vegetable production. According to the relative scale between the reflective board and the shelf setting in IFR used in this study, the proportion of board width and distance between the light source and the tray surface is suggested to be 10:12 to achieve the optimal growth and quality improvement of plant. The ALR can be adapted further for diverse growth systems and plant species. Future studies should be focused on the effects of combined ALR and red/blue lights as the light source and photoperiod as well on the growth and quality of vegetable seedlings in order to further reduce power consumption and enhance the energy conversion efficiency in indoor farming.

## Nomenclature

%RH, relative humidity; ALDA, available light distribution area; ALR, adjustable lampshade-type reflector; AWA, acetone–water–acetic acid; Chl*a*, chlorophyll *a*; Chl*b*, chlorophyll *b*; DPPH, 2,2-diphenyl-1-picrylhydrazyl; DW, dry weight; FCR, Folin–Ciocalteu’s phenol reagent; FW, fresh weight; GAE, gallic acid equivalents; GUI, graphical user interface; HD, hypocotyl diameter; HI, harvest index; HL, hypocotyl length; IFR, indoor farm racks; LDU, light distribution uniformity; LED, light-emitting diode; PAR, photosynthetically active radiation; PE, photosynthetic efficiency; PPED, photosynthetic photon energy density; PPFD, photosynthetic photon flux density; RL, root length; ROS, reactive oxygen species; SD, standard deviation; TC, total carotenoids; Tchl, total chlorophylls; TE, Trolox equivalents; TLA, total leaf area; TPC, total phenolic content.

## Data availability statement

The original contributions presented in the study are included in the article/supplementary materials, further inquiries can be directed to the corresponding author/s.

## Author contributions

JH: conceptualization, methodology, investigation, data collection and processing, writing—original draft, and writing—review and editing. ZG: data collection and processing and writing—original draft. XH: data collection and processing and writing—original draft. WZ: conceptualization, methodology, writing—review and editing, supervision, and project administration. All authors contributed to the article and approved the submitted version.
